# Evaluation of Two Shaping Systems and Two Ultrasonic Irrigation Devices in Removing Root Canal Filling Material from Mesial Roots of Mandibular Molars: A Micro CT Study

**DOI:** 10.3390/dj7010002

**Published:** 2019-01-02

**Authors:** Marc Krikor Kaloustian, Walid Nehme, Claire El Hachem, Carla Zogheib, Nabil Ghosn, Jérôme Michetti, Alfred Naaman, Franck Diemer

**Affiliations:** 1 Department of Endodontics, Faculty of Dentistry, Saint Joseph University, Beirut Po 11 5070, Lebanon; walidnehmeendo@gmail.com (W.N.); zogheibcarla@gmail.com (C.Z.); alfrednaaman@gmail.com (A.N.); 2 Department of Pediatric Dentistry, Faculty of Dentistry, Saint Joseph University, Beirut Po 11 5070, Lebanon; claire.elhachem@gmail.com; 3 Department of Oral and Maxilla-Facial Radiology, Faculty of Dentistry, Saint Joseph University, Beirut Po 11 5070, Lebanon; nabil.ghosn@usj.edu.lb; 4 Institut de Recherche en Informatique de Toulouse, IRIT, CNRS UMR 5505, 118 Route de Narbonne, 31062 Toulouse CEDEX 9, France; Jerome.michetti@gmail.com; 5 Faculté de Chirurgie Dentaire CHU de Toulouse, Institut Clement Ader (labo), 118 Route de Narbonne, 31062 Toulouse CEDEX 9, France; franck.diemer@wanadoo.fr

**Keywords:** retreatment, Reciproc, 2Shape, passive ultrasonic irrigation, micro-CT

## Abstract

We assessed the efficiency of two shaping file systems and two passive ultrasonic irrigation (PUI) devices for removing filling material during retreatment. The mesial canals from 44 extracted mandibular molars were prepared and obturated. The teeth were randomly divided into two groups, and then one group was retreated with Reciproc R25 (VDW, Munich, Germany) (n = 44) and the other group was retreated with 2Shape (TS, Micro Mega, Besançon, France) (n = 44). A micro-computed tomography (CT) scan was taken before and after the retreatment to assess the volume of the filling material remnants. The teeth were then randomly divided into four groups to test two different PUI devices: Irrisafe (Satelec Acteon Group, Merignac, France) and Endo Ultra (Vista Dental Products, Racine, WI, USA). The teeth in Group A were retreated with 2Shape to test the Endo Ultra (n = 22) device, the teeth in Group B were retreated with 2Shape in order to test the Irrisafe (n = 22) device, the teeth in Group C were retreated with Reciproc to test the Endo Ultra (n = 22) device, and Group D was retreated with Reciproc to test the Irrisafe (n = 22) device. A third micro-CT scan was taken after the retreatment to test the PUIs. The percentage of Gutta-Percha (GP) and sealer removed was 94.75% for TS2 (*p* < 0.001) and 89.3% for R25 (*p* < 0.001). The PUI significantly enhanced the removal of the filling material by 0.76% for Group A (*p* < 0.001), 1.47% for Group B (*p* < 0.001), 2.61% for Group C (*p* < 0.001), and by 1.66% for Group D (*p* < 0.001). 2Shape was more effective at removing the GP and sealer during retreatment (*p* = 0.018). The supplementary approach with PUI significantly improved filling material removal, with no statistical difference between the four groups (*p* = 0.106).

## 1. Introduction

Root canal treatments have a high success rate [1], but failures occasionally occur [2]. In this case, nonsurgical root canal retreatment is considered as the first treatment option [3]. The favorable outcome of retreatment depends, amongst other criteria, on the complete elimination of the previous root filling material that may harbor bacteria and its by-products [4].

Many techniques have been advocated for Gutta-Percha (GP) and sealer removal, including the use of solvents, heat, and various instruments, such as stainless steel hand files and nickel-titanium (NiTi) files [5,6].

In reciprocation, Reciproc (VDW, Munich, Germany), originally designed for shaping procedures, yielded positive results when used in retreatment [7].

In continuous rotation, a new shaping system, 2Shape (TS, Micro Mega, Besançon, France) was introduced. The system includes two files, TS1 and TS2, with a 25 tip, with a 4% and 6% taper, respectively. The files were heat-treated and they displayed a unique cross-section, with a modified triple helix presenting an off-centered blade. To the best of our knowledge, no reports regarding the efficacy of this system in retreatment have been published. Studies conducted so far have proven that no sole file system is able to completely remove the filling material [8,9]. Additional techniques, such as passive ultrasonic activation (PUI), have been suggested to eliminate the remaining material after instrumentation, but the results are inconsistent [10,11,12]. Irrisafe (Satelec Acteon Group, Merignac, France) is an ultrasonic device that works in the range of 25 to 30 kHz, which activates the irrigation solution by acoustic streaming and micro-cavitation [13,14]. Endo Ultra (Vista Dental Products, Racine, WI, USA) is a new wireless ultrasonic device. According to the manufacturer, it is the only device that is capable of generating a frequency of 40 kHz and able to effectively clean and remove vapor lock. To date, no studies have compared the usage of both Endo Ultra and Irrisafe in retreatment.

High-resolution micro-computed tomography (micro-CT) is largely used to evaluate the debris left inside root canals, as it is a non-destructive and reproducible method [7].

The aim of this study was to compare Reciproc R25 retreatment to TS2 retreatment in removing Gutta Percha (GP) and sealer from the mesial canals of mandibular molars. We investigated whether the additional use of the Irrisafe and Endo Ultra PUIs improved cleanliness, and whether there are differences between the two PUIs.

The first null hypothesis is that there is no significant difference between the TS2 and R25 retreatment results in removing a root canal filling from the mesial roots of mandibular molars. The second null hypothesis is that PUI usage does not have any additional effect in improving the removal of the GP and sealer. 

## 2. Materials and Methods

### 2.1. Sample Selection

Approval was obtained from the institutional ethical committee of the Saint Joseph University (USJ-2017-55). Two hundred mandibular molars, extracted for reasons that are independent from our study, were cleaned with an ultrasonic tip (1S, Satelec Acteon Group, Merignac, France) and stored in 0.1% formocresol. The samples were inspected under a dental operating microscope (Zeiss Extaro 300, Oberkochen, Germany) at 16× magnification in order to eliminate teeth with cracks or advanced external resorption. Mesio-distal and bucco-lingual radiographs (Sopix, Satelec Acteon Group, Merignac, France) were taken and the samples with previously treated canals, pulp calcification, or internal resorption were discarded. A specialist in endodontics performed all root canal preparation, including the filling and retreatment procedures, under 16× magnification. After the access cavity preparation, two #10/0.02 files (Kendo CC cord, VDW, Munich, Germany) were simultaneously introduced into the canals to the foramen in order to check the patency of the two separate exits. Only teeth that presented with fully formed apexes and separate mesial canals, with a moderate curvature ranging from 15 to 22 degrees according to Schneider’s technique, were included in the study [15,16]. Forty-four molars met the inclusion criteria (n = 88).

Crowns were cut into the teeth with a diamond disc (kerr Dental, Bioggio, Switzerland) until a 17 mm root length was reached. A 1 mm plexiglass was sealed around the access cavity of each crown, using hot glue and a silicone key that was produced for consistent scan positioning.

### 2.2. Initial Preparation and Obturation

Both mesial canals were shaped with the ProTaper Gold rotary system (Dentsply/Maillefer, Ballaigues, Switzerland) according to the manufacturer’s instructions: the coronal third was shaped with the SX file; the S1, S2, and F1 files were used to the set working length (WL), at 1 mm shorter than the apical foramen. Apical patency was checked between each instrument with a #10K-file and the canal was irrigated with 3 mL 6% sodium hypochlorite (NaOCL), with a 30-G NaviTip needle (Ultradent, South Jordan, UT, USA). The smear layer was removed using 3 mL 17% EDTA, followed by a final rinse with 3 mL 6% NaOCL. Each of the instruments was used to prepare four canals.

The canals were dried using sterile medium paper points and were obturated with fine GP points (Hygienic, Coltene Whaledent, Langeneau, Germany), pulp canal sealer EWT (Sybron Dental Specialties, Orange, CA, USA), and the continuous wave vertical compaction technique, using system B (Sybron Dental Specialties, Orange, CA, USA) with a Medium Fine (MF) 0.04 plugger (Sybron Dental Specialties, Orange, CA, USA). Additional GP points and a Gutta condenser (40/0.02) (Dentsply/Maillefer, Ballaigues, Switzerland) were used for the backpack.

The access cavities were sealed with temporary filling material (Cavit, 3M ESPE, Seefeld, Germany).

Digital radiographs were taken, mesio-distally and bucco-lingually, to verify the density of the obturation. Teeth were then stored at 37 °C in 100% humidity for 14 days to allow the sealer setting.

### 2.3. Pre-Retreatment Micro-CT Imaging

Tooth imaging was performed on a micro-CT platform (EA2496, Hopkinton, Montrouge, France). Each tooth was individually scanned with a micro-CT scanner (Quantum FX; PerkinElmer Health Sciences, Hopkinton, MA, USA) after the root canal obturation. The field of view was set to 10 mm to obtain three-dimensional (3D) images with an isotropic resolution of 20 µm. The acquisition settings were 160 kV, 90 mA, and 360° scanning rotation.

### 2.4. Retreatment Procedures

OneFlare (25/0.09) (Micro Mega, Besançon, France) was used 2 mm inside the canals to initiate the retreatment procedure in the coronal part of the teeth. This orifice opener is the first instrument that used T-wire heat treatment according to the manufacturer. By exposure to T-wire heat treatment after the grinding process, the microstructure and mechanical properties of the NiTi alloy change slightly. Better flexibility and cyclic fatigue resistance are observed in heat-treated NiTi alloy as compared with non-heat-treated instruments. It has a 25 tip with a 9% taper up to 8 mm, then no taper at all because the wire used has a 1 mm diameter [17].

The canals were then randomized using a computer algorithm according to the retreatment technique and were allocated into two groups: TS2 and R25 (n = 44). In Group 1, TS2 (25/0.06) was used on a MM (Micro Mega, Besançon, France) control motor at 300 rpm and 2.5 Ncm in a pecking and brushing motion, with an amplitude of 3 mm. After three movements, the instrument was cleaned with sterile gauze, inspected, and reinserted until the WL was reached. In Group 2, R25 (25/0.08) was used on a VDW Silver Reciproc motor in a pecking and brushing motion, with an amplitude of 3 mm. After three movements, the instrument was cleaned with sterile gauze, inspected and the procedure was repeated until the WL was reached.

For both groups, each set of instruments was used to retreat two canals. For the irrigation protocol, each canal was washed with 12 mL 6% NaOCL with a 30-G NaviTip needle (Ultradent, South Jordan, UT, USA). Subsequently, 3 mL 17% EDTA was left inside the canal for 1 min and another 3 mL 6% NaOCL was used as a final rinse. The retreatment was considered complete when the WL was reached, with no residual filling material being detected on the root canal walls under clinical microscope (16×) or on the flutes of the instruments.

### 2.5. Post Retreatment Micro-CT Imaging

After retreatment with both systems, the roots were scanned using the micro-CT device with the same parameters mentioned above. The volume and percentage of the filling material remnants were calculated for each group in the coronal, middle, and apical third.

### 2.6. Supplementary Approach Using Passive Ultrasonic Irrigation

To test the two PUI devices, the canals were divided into four groups (n = 22 each):

(1) Group A: TS2 retreatment with the Endo Ultra PUI
(2) Group B: TS2 retreatment with the Irrisafe PUI
(3) Group C: R25 retreatment with the Endo Ultra PUI
(4) Group D: R25 retreatment with the Irrisafe PUI

The Irrisafe (20/0.02) and Endo Ultra (20/0.02) devices activated the irrigation process in the same sequence 1 mm from the WL. Subsequently, irrigation using 3 mL 6% NaOCL followed by 20 s activation, was repeated three times. The canals were dried and irrigated with 3 mL 17% EDTA, followed by 1 min activation and a final rinse using 3 mL 6% NaOCL. 

### 2.7. Post PUI Micro-CT Imaging

A third micro-CT scan with the same parameters was taken to assess the effect of the two additional PUI devices on the removal of the GP and sealer.

### 2.8. Micro-CT Analysis

Three-dimensional images were reconstructed using Quantum FX micro-CT^®^ software (March 2013, PerkinElmer Health Sciences, Hopkinton, MA, USA). An automatic rigid registration was achieved using the MATLAB^®^ software (MATLAB 9.3, MathWorks, Natick, MA, USA) to align the three stages of acquisition for comparison. A global thresholding technique, which is based on Otsu’s method [18], was applied to segment the hard tissue of the teeth, the background, and the filling material remnants. Volumes were then binarized to retain only the remnants, and the preoperative and postoperative filling material volumes were quantified and expressed in mm^3^ [19]. The localization of the residual filling material was performed according to the canal thirds. The percentage of remaining filling material after retreatment was calculated, as follows: A/B × 100 = Volume (%) of the remaining filling material, where A represents the postoperative volume of the filling material and B represents the preoperative volume of the filling material.

### 2.9. Statistical Analysis

The data was analyzed using the SPSS software for Windows (version 24.0, Chicago, IL, USA). The level of significance was set at 0.05 (*p* < 0.05). The normality distribution of the continuous variables was assessed with the Kolmogorov-Smirnov test. Since the variables were not normally distributed, non-parametric tests were performed. The Wilcoxon test was used to compare the volume of the filling material before and after retreatment and the volume of the residual material before and after PUI usage. The Mann-Whitney test was used to compare the percentage of the residual material after retreatment between the two systems. The Kruskal-Wallis tests was used to compare the percentage of residual product remaining, after activation, between the four groups.

## 3. Results

Three TS2 instruments were deformed, whereas none were deformed in the R25 group. In the entire mesial canal, the volume of the filling material dropped significantly from 2.51 mm^3^ to 0.13 mm^3^, with TS2 and from 2.34 mm^3^ to 0.24 mm^3^ with R25.

The percentage of the filling material removed was 94.75% with TS2 (*p* < 0.001); 89.3% was removed with R25 (*p* < 0.001), with a significant difference between the two groups (*p* = 0.018). In the entire canal, the PUI decreased the residual volume of the filling material after retreatment by 0.76% for group TS2 with Endo Ultra (*p* < 0.001), 1.47% for group TS2 with Irrisafe (*p* < 0.001), 2.61% for group R25 with Endo Ultra (*p* < 0.001), and by 1.66% for the group R25 with Irrisafe (*p* < 0.001) (Table 1). No statistically significant difference was observed between the four groups (*p* = 0.106).

**Figure 1:**
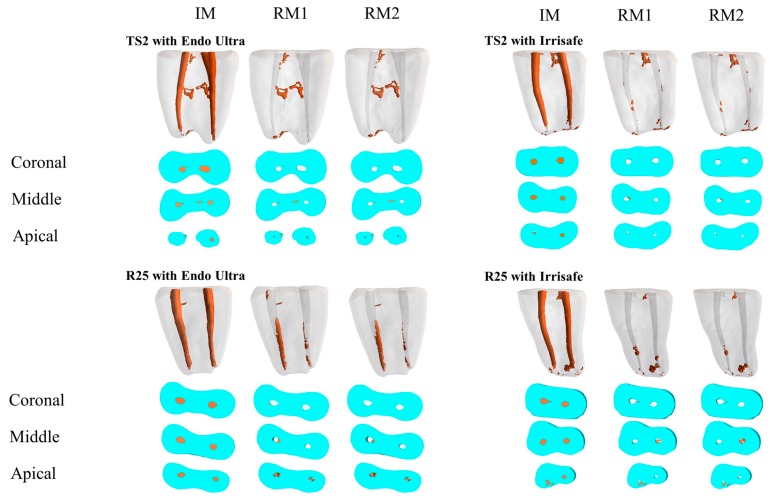
Reconstructed three-dimensional micro-computed tomography (3D μ-CT) images and cross-sections for groups TS2 with Endo Ultra, TS2 with Irrisafe, R25 with Endo Ultra, and R25 with Irrisafe, including initial material (IM), residual material (RM1), and the residual material after passive ultrasonic activation (PUI) (RM2), with the corresponding technique.

The postoperative percentage of residual material in the entire canal (after retreatment and activation) was significantly smaller in Groups A (3.45%) and B (4.87%) when compared with Groups C (9.42%) and D (7.54%) (*p* = 0.040) (Figure 1).

## 4. Discussion

In this study, we aimed to assess the efficiency of two different shaping systems and two ultrasonic activation devices that are used in retreatment by evaluating the residual material with the aid of micro-CT. The first null hypothesis was rejected because a significant difference was observed between the instruments in the removal of filling materials from the mesial roots of mandibular molars. The second null hypothesis regarding the additional activation techniques was also rejected, since PUI improved the removal of GP and sealer (*p* > 0.05).

None of the instruments were able to completely remove the filling material during retreatment, which is in accordance with previous studies [20,21]. As found by Bernades et al. [21], Reciproc is associated with less retained filling material in canals than ProTaper and manual techniques. The difference that was observed in the results might be attributed to a difference in the protocols used in these experiments. Nevertheless, our results showed that TS2 is more effective than R25. With the use of TS2, there was less residual material in the canal (*p* = 0.018). These results may be attributed to two factors: file design and alloy processing.

Reciproc is made from M wire and should have higher strength and wear resistance due to the unique nano-crystalline microstructure. The Austeniste Finish (AF) temperature of Reciproc is around 50 °C, conferring more flexibility to the file due to the higher transformation temperature [22,23]. TS2 undergoes a different thermomechanical process, including post machining electro polishing, followed by heat treatment, which is a proprietary process undisclosed by Micro Mega (Besançon, France). According to the manufacturer, T-wire heat treatment results in instruments that have better flexibility and cyclic fatigue resistance when compared with instruments that were manufactured using the traditional austenite NiTi alloy [24]. 

Despite of the gold color of TS2, in a separate pilot study using a differential scanning calorimetric test, the file was found to have an AF temperature of 17 °C. Therefore, it is an austenitic file. The lower AF might confer greater stiffness to the file, as well as increasing its ability to remove filling material [23]. The stiffness of the file might also be related to the pitch. TS2 has greater pitch than Reciproc and increasing the pitch would increase the stiffness of the file according to Versluis et al. [25].

The design of the file, with its helical angles and cross section, could also play a role in its flexural stiffness and potential for debris eviction [22]. Small helical angles and variable pitch length might contribute to the fast eviction of debris toward the shaft of the files. Reciproc shows a column cross-section and a regressive taper, starting with 8% [26]. TS2 presents a modified triple helix cross-section with an off-centered blade and a continuous taper of 6%, allowing for the file to better contact canal walls and potentially remove more debris by creating a final shape larger than the taper of the file itself [10]. This is a claim already stated by the manufacturer, highlighting its ability to improve cutting efficiency and debris removal [24].

The superior performance of the 2Shape system could be related to the continuous rotation movement, which produces a constant flow of debris in the coronal direction; the reciprocating movement might displace the debris apically [27]. However, this interpretation is considered to be controversial if consulting the results of other studies [28].

In this study, the sample preparation protocol followed that used in a previous study [20]. Mandibular molars classified as Vertucci type IV configuration were chosen. This root morphology is particularly interesting in comparing two treatment/retreatment techniques, because it presents two root canals that are very similar in terms of anatomy, allowing the comparison of two techniques by alternating the two systems in the mesial canals from one sample to another. Micro-CT evaluation is preferred because of several advantages, including its capacity to evaluate the presence of root canal filling remnants during different stages of the experiment without the need to destroy the specimen [12]. The same image quality cannot be attained with other available imaging technologies [7]. Serial analysis of the samples cannot be performed with clearing or sectioning techniques [5,9]. Furthermore, all of the retreatment procedures were performed under microscope (16×) in order to eliminate as much GP and sealer as clinically possible [12,29,30].

We chose to not use solvents in this study because the aim was to assess the mechanical properties of the instruments and some studies found that the use of solvents did not improve the effectiveness of the files in retreatment [5]. Solvents could enhance the root canal penetration of files, but it may hinder cleaning by creating a layer of softened GP that forms and adheres to the root canal walls [31].

The final size of the preparation with TS2 (25/0.06) and with a variable taper with R25 (25/0.08) was established based on the size of the previous filling (20/0.07) to mimic the clinical reality of underprepared canals. This did not have an impact on the cleanliness of the canals. Larger sizes were used in similar studies using different samples, such as maxillary central incisors or mandibular incisors [21,32]. Some authors found that enlarging the rotary files by two sizes beyond the initial preparation size resulted in a reduction in the amount of residual filling material [33,34], whereas others concluded that the use of larger rotary files might cause severe transportation of the apical part of the canal while failing to clean the wall on the inner side [35].

In the present study, the results showed that instrumentation alone was not able to completely remove the filling material from within root canals, which corroborates the findings of previous studies [8,11,36]. In some studies, agitation of the irrigating solutions using sonic and ultrasonic methods has been advocated to enhance the removal of filling material remnants [21,37]. In other studies, no significant difference was found in terms of filling material removal before and after PUI [38,39]. The differences in root canal morphology, the type of filling material, and techniques that were used for retreatment could possibly explain the conflicting results. In this study, PUI devices significantly improved filling material removal. These results are similar to those that were obtained by Friedman et al. [40] and Cavenago et al. [37].

After applying the PUI, a combination of TS2 and a PUI leads to fewer remnants in the whole canal (*p* = 0.040). This might be explained by the fact that less residual material remained after the initial use of TS2. Therefore, less contact could have occurred between the ultrasonic tip and the canal walls, allowing its free vibration and the removal of the residual filling material. 

The limitations of this study lie in its in vitro design. More studies with different anatomical variations and other filling materials should be performed in order to confirm the results. This is the first study to evaluate T-wire technology in retreatment, so additional studies should be completed to confirm the effectiveness of these instruments in removing GP and sealer.

In conclusion, TS2 retreatment is more effective than the R25 retreatment in eliminating GP and sealer. The supplementary approach with the use of a PUI significantly improved filling material removal, without a statistically significant difference between the Irrisafe and Endo Ultra devices. More studies should be conducted to find the perfect combination of instrumentation and PUI for the optimal removal of filling material during the retreatment procedure.

## Figures and Tables

**Table 1:** The mean volume and standard deviation (mm^3^) of the initial material (IM), residual material after the retreatment procedure (RM1), residual material after the additional cleaning method (RM2), and the mean percentage of the residual filling material (%) after applying the additional cleaning methods, according to the groups studied.

Groups	n	IM	RM1	RM2	%
TS2 with Endo Ultra	22	2.50 ± 0.37	0.10 ± 0.07	0.08 ± 0.05	3.45 ± 2.62
TS2 with Irrisafe	22	2.52 ± 0.36	0.16 ± 0.13	0.13 ± 0.125	4.87 ± 4.66
R25 with Endo Ultra	22	2.34 ± 0.49	0.27 ± 0.22	0.20 ± 0.18	9.42 ± 8.22
R25 with Irrisafe	22	2.35 ± 0.36	0.22 ± 0.22	0.18 ± 0.18	7.54 ± 6.96
